# Clinical outcomes of non‐osteogenic, non‐Ewing soft‐tissue sarcoma of bone––experience of the Toronto Sarcoma Program

**DOI:** 10.1002/cam4.3531

**Published:** 2020-10-16

**Authors:** Zachary W. Veitch, Samir Fasih, Anthony M. Griffin, Esmail M. Al‐Ezzi, Abha A. Gupta, Peter C. Ferguson, Jay S. Wunder, Albiruni R. Abdul Razak

**Affiliations:** ^1^ Toronto Sarcoma Program at Mount Sinai Hospital Toronto Canada; ^2^ Princess Margaret Cancer Centre Toronto Canada; ^3^ The Hospital for Sick Children Toronto Canada; ^4^ University of Toronto Musculoskeletal Oncology Unit Mount Sinai Hospital Toronto Canada; ^5^ Division of Orthopaedic Surgery Department of Surgery University of Toronto Toronto Canada

**Keywords:** bone sarcoma, osteosarcoma, Sarcoma, soft‐tissue sarcoma, Toronto Sarcoma

## Abstract

Non‐osteogenic, non‐Ewing soft‐tissue sarcoma (NONE‐STS) of bone is a rare presentation of primary bone cancers. Optimal treatments and outcomes for this heterogenous group are poorly described. We evaluated the factors associated with long‐term outcomes in patients with this disease. Patients with localized NONE‐STS of bone treated at the Toronto Sarcoma Program from 1987 to 2017 were identified. Clinical characteristics, treatment, and survival information were collected. Kaplan‐Meier (log‐rank) survival estimates from the time of definitive surgery, with uni‐/multivariate analyses (Cox) of sarcoma‐specific survival were performed. A total of 106 patients (60.4% male; median age 46 years) with NONE‐STS of bone were identified. Common histologies included undifferentiated pleomorphic sarcoma [UPS]/malignant fibrous histiocytoma [MFH] (UPS/MFH, 41.5%), leiomyosarcoma (LMS, 20.8%), and fibrosarcoma (FS, 11.3%). Tumors were often high grade (59.4%) and involved the extremities (88.7%), with most receiving chemotherapy (67.9%) with cisplatin/doxorubicin‐based regimens (73.6%). In the full cohort, 10‐year DFS (45.7%, [95%CI: 35.7‐55.8%]), OS (53.4%, [95%CI: 41.7‐62.2%]), and SSS (63.9%, [95%CI: 53.9‐72.5%]) were moderate. Histology specific, 10‐year SSS was 70.7% [95%CI: 56.1‐85.5%] for UPS/MFH, 51.8% [95%CI: 29.8‐73.8%] for LMS, and 72.2% [95%CI: 45.1‐99.2%] for FS. Only UPS/MFH (n = 4) showed sarcoma‐related death >10 years. Multivariate analysis identified axial location (HR = 35.5, [95%CI: 3.4‐369.6]), high grade (HR = 16.9, [95%CI: 1.6‐185.1]), and disease relapse (HR = 485.1, [95%CI: 36.3‐6482.6]) as risk factors for death (*p* < 0.05). Treatment with chemotherapy (HR = 0.1, [95%CI: 0.01‐0.86]) and necrosis ≥85% (HR = 0.2, [95%CI: 0.04‐0.99]) showed improved survival (*p* < 0.05). NONE‐STS of bone has favorable long‐term survival similar to osteosarcoma. Patients receiving chemotherapy derive benefit in retrospective analyses. UPS/MFH histologies show sarcoma‐related death beyond 10 years. Further data on histologic subgroups are needed.

## Graphical abstract

In patients with Non‐osteogenic, non‐Ewing soft‐tissue sarcoma (NONE‐STS) of bone, long‐term survival is similar to that of osteosarcoma. Patients receiving chemotherapy who demonstrated ≥85% necrosis on pathologic specimens had improved survival, whereas those with UPS/MFH histology experienced sarcoma‐related death beyond 10 years.

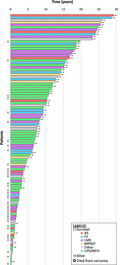

## 1 INTRODUCTION

Non‐osteogenic, non‐Ewing soft‐tissue sarcoma (NONE‐STS) of bone includes a heterogenous group of sarcoma histologies including undifferentiated pleomorphic sarcoma [UPS] (formerly known as malignant fibrous histiocytoma [MFH]), leiomyosarcoma [LMS], fibrosarcoma [FS], angiosarcoma [AS], and malignant peripheral nerve sheath tumors [MPNST], among others.^1^, ^2^ The optimal management of this atypical presentation of soft‐tissue sarcoma as a primary bone tumor is poorly described due to its infrequent nature. NONE‐STS of bone are generally treated similar to high‐grade osteosarcoma with wide surgical resection in addition to cisplatin/doxorubicin‐based neoadjuvant, adjuvant, or perioperative (both prior to, and after definitive surgery) chemotherapy as per North American and European guidelines.^3^, ^4^

In UPS of bone, approximately one‐quarter occur in relation to prior bone insults (e.g.: radiation, Paget's disease of the bone, osteonecrosis, and prosthetic hip surgery) or heritable conditions such as Hardcastle syndrome (diaphyseal medullary stenosis).^5^ Chemosensitivity and 5‐year survival of UPS of bone is thought to be similar to osteosarcoma^6^, ^7^; although late recurrences out to 10 years have been documented.^8^ Outcomes for patients with nonmetastatic LMS of bone are generally favourable,^9^, ^10^ whereas outcomes for patients with FS treated prior to modern chemotherapy and surgical techniques are thought to be relatively poor.^11^, ^12^ Evaluation of AS of bone by the European Musculoskeletal Oncology Society (ESMOS) found moderate 5‐year survival for patients with localized disease, albeit with short median follow‐up.^13^ Survival for MPNST of bone is generally unknown and described only in case reports.^14^, ^15^, ^16^ In a study by Berner et. al evaluating spindle cell sarcomas (e.g.: UPS, FS, and LMS) of bone using Norwegian Cancer Registry data over a 34‐year period, a low 5‐year sarcoma‐specific survival (SSS) in a mixed metastatic and localized disease cohort was shown.^1^ Additional information to guide clinical discussion and prognoses is urgently needed for this uncommon presentation of NONE‐STS of bone.

The Toronto Sarcoma Program is a large, multidisciplinary treatment program based at a quaternary cancer treatment facility (Mount Sinai Hospital) in Toronto, Canada. In this context, we aimed to identify grouped‐ and histology‐specific survival outcomes in addition to patient and treatment characteristics associated with survival in NONE‐STS of bone.

## 2 METHODS

### 2.1 Patient selection

Patients with sarcoma of the bone treated at the Toronto Sarcoma Program from 01/1987 to 12/2017 were identified from the University of Toronto Musculoskeletal Oncology sarcoma database (Mount Sinai Hospital, Toronto, Canada). Patients with a diagnosis of osteosarcoma, Ewing sarcoma of bone, or metastatic disease at diagnosis were excluded. For patients with localized non‐osteogenic, non‐Ewing (NONE) sarcoma of bone, patient and tumor characteristics (age, sex, histology, tumor size, histologic grade, chemotherapy, and/or radiotherapy induced tumor necrosis, surgical margins, tumor location, and relapse), treatment modality (surgical, chemotherapy [sequence and regimen] and radiotherapy), postoperative complications, and survival information were retrospectively collected. Ambiguous sarcoma pathologies were determined after internal consensus pathology review at time of diagnosis as per institutional standards. Retrospective pathologic re‐adjudication was not performed on tumor specimens, and patients with a historical designation of MFH were grouped with UPS reflecting changes in nomenclature over time.^17^ Degree of tumor necrosis was adjusted and stratified as <85% vs ≥85% based on prior studies in soft tissue sarcoma.^18^, ^19^, ^20^, ^21^ As per institutional standard of care, all patients were reviewed at Mount Sinai Hospital sarcoma tumor boards. This study was approved by the institutional research ethics board.

### 2.2 Statistics

Clinical and treatment characteristics were reported descriptively. For survival calculations, time from definitive surgery to event of interest was used for disease‐free survival (DFS) [recurrence or death], overall survival (OS) [death from any cause], and SSS, [sarcoma‐related death]. Survival estimates for DFS, OS, and SSS were performed using the Kaplan‐Meier method (with log‐rank analyses where appropriate). A Cox proportional hazards model was used for SSS estimates in univariate and multivariate analysis of clinical and treatment characteristics. Hazard ratios (HR) with 95% confidence intervals (95% CI) are reported. For all tests, *p* ≤ 0.05 was considered significant. Statistical analyses were performed using IBM SPSS Statistics v24 (IBM) and XLSTAT v2019.1 (Addinsoft).

## 3 RESULTS

### 3.1 Patient selection and characteristics

**FIGURE 1:**
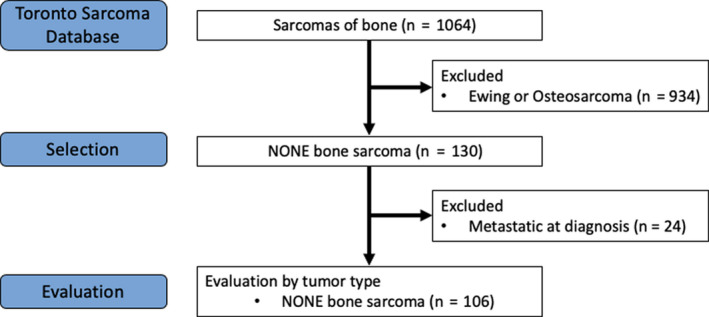
Consort diagram of patient selection. Abbreviations: NONE, non‐osteosarcoma, non‐Ewing

A total of 1064 patients with sarcoma of the bone treated over a 30‐year period were identified (Figure 1). Of 130 patients with NONE‐STS of bone, 106 without distant metastases at diagnosis met study inclusion criteria. The majority of patients were male (60.4%) with a median age of 46 (range 18‐89) years and had sarcoma of the lower extremity (79.2%) (Table 1). Predominant NONE‐STS of bone histologies were UPS/MFH (41.5%), LMS (20.8%), and FS (11.3%), with other (12.3%) comprised of solitary fibrous tumor (n = 1), spindle cell tumor (n = 2), sarcoma NOS (n = 3), mixed sarcoma (n = 1), mesenchymoma (n = 2), epithelioid hemangioendothelioma (n = 2), and malignant giant cell tumor (n = 2).

**TABLE 1:** Clinical, treatment, and survival characteristics for NONE‐STS of bone.

Patient Characteristics (N = 106)			
**Median age [range]**	46 [18‐89]	**Pathology**	**N (%)**
**Sex**	**N (%)**	UPS/MFH	44 (41.5)
Male	64 (60.4)	Leiomyosarcoma	22 (20.8)
Female	42 (39.6)	Fibrosarcoma	12 (11.3)
**Location**		Angiosarcoma	9 (8.5)
Lower	84 (79.2)	MPNST	6 (5.7)
Upper	10 (9.4)	Other	13 (12.3)
Axial	12 (11.3)	**Tumor Size**	
**Surgery Type**		≤5 cm	13 (12.3)
Amputation	21 (19.8)	>5‐10 cm	51 (48.1)
Limb Sparing	85 (80.2)	>10 cm	26 (24.5)
**Postoperative Complication**		Unknown	16 (15.1)
Yes	35 (33.0)	**Grade**	
No	71 (67.0)	1	13 (12.3)
***Postoperative Complication Type***		2	20 (18.9)
Wound Debridement	12 (34.3)	3	63 (59.4)
Revision	10 (28.6)	Unknown	10 (9.4)
Fracture	6 (17.1)	**Surgical Margin**	
Infection	3 (8.6)	Negative	90 (84.9)
Other	4 (11.4)	Positive	11 (10.4)
**Radiation**		Unknown	5 (4.7)
Yes	7 (6.6)	**Necrosis**	
No	99 (93.4)	0‐49%	27 (40.3)
**Chemotherapy**		50‐84%	12 (17.9)
Yes	72 (67.9)	85‐100%	21 (31.3)
No	34 (32.1)	Unknown	7 (10.4)
**Chemotherapy Regimen**		No Pre‐op Treatment	39 (36.8)
Cis/Dox	37 (51.4)	**Disease Recurrence**	
Cis/Dox/MTX +Etopo/Ifos	8 (11.1)	Yes	44 (41.5)
Cis/Dox/MTX/Ifos	6 (8.3)	No	62 (58.5)
Cis/Dox/MTX	2 (2.8)	***Recurrence Location***	
Other	5 (6.9)	Local	8 (18.2)
Unknown	14 (19.4)	Systemic	36 (81.8)
**Number of Chemotherapy Cycles**		*Lung*	24 (66.7)
2‐4	8 (11.1)	Bone	11 (30.6)
5‐6	41 (56.9)	Nodes	3 (8.3)
7‐8	2 (2.8)	Soft Tissue	2 (5.6)
Unknown	21 (29.2)	Liver	1 (2.8)
**Neoadjuvant Treatment**		Unknown	4 (11.1)
Chemotherapy +/− Radiotherapy	65 (97.0)	**Survival**	
Radiotherapy only	2 (3.0)	Alive	54 (50.9)
—	—	Deceased	52 (49.1)
—	—	*Sarcoma‐related death*	37 (71.0)

Abbreviations: MPNST, malignant peripheral nerve sheath tumor; UPS, undifferentiated pleomorphic sarcoma; MFH, malignant fibrous histiocytoma; NONE, non‐osteosarcoma, non‐Ewing; MTX, methotrexate; Etopo, Etoposide; Ifos, Ifosfamide

Patients receiving chemotherapy (67.9%) commonly had neoadjuvant (n = 21), adjuvant (n = 7), or perioperative chemotherapy (n = 44) administered as a cisplatin/doxorubicin‐doublet (51.4%), or in combination with additional cytotoxic agents (22.2%) [e.g.: methotrexate, ifosfamide, etoposide] (Table 1). The median number of chemotherapy cycles was 6 (range 2‐8). Only seven patients (6.6%) received adjuvant (n = 2) or neoadjuvant (n = 5) radiotherapy as a single modality (n = 3) or in addition to (n = 4) chemotherapy. Of 13 patients with grade 1 tumors, 3 (2.8%) received chemotherapy.

Limb sparing surgical procedures were performed for 80.2% of patients with NONE‐STS of bone with pathology of resected tumors showing a predominance toward high grade (59.4%), negative surgical margins (84.9%), <50% necrosis (40.3%) for patients receiving neoadjuvant treatment (n = 67), and tumor size >5 cm (72.6%) on final pathology. Postoperative complications occurred in 33% of patients, with reasons for complications including need for debridement (n = 12), surgical revision (n = 10), fracture (n = 6), infection (n = 3), and other (n = 4). Disease recurrences were frequently systemic (81.8%), with lung (66.7%), bone (30.6%), and lymph nodes (8.3%) as common sites. Of 44 total recurrences, only 8 were local (18.2%).

### 3.2 Survival outcomes

**FIGURE 2:**
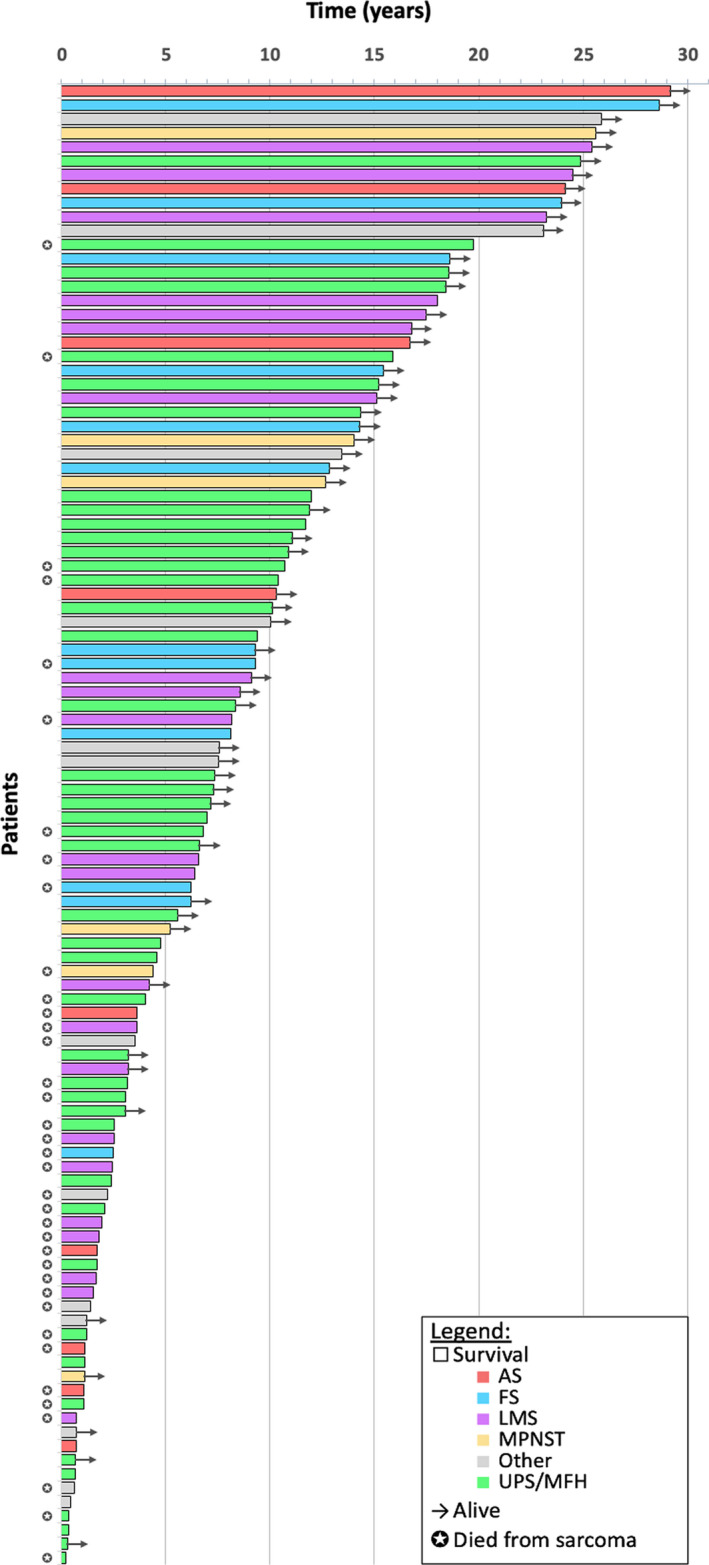
Swimmers plot of patient survival by histology for NONE‐STS of bone from time of definitive surgery. Abbreviations: AS, angiosarcoma; FS, fibrosarcoma; LMS, leiomyosarcoma; MPNST, malignant peripheral nerve sheath tumor; UPS, undifferentiated pleomorphic sarcoma; MFH, malignant fibrous histiocytoma; NONE, non‐osteosarcoma, non‐Ewing

**FIGURE 3:**
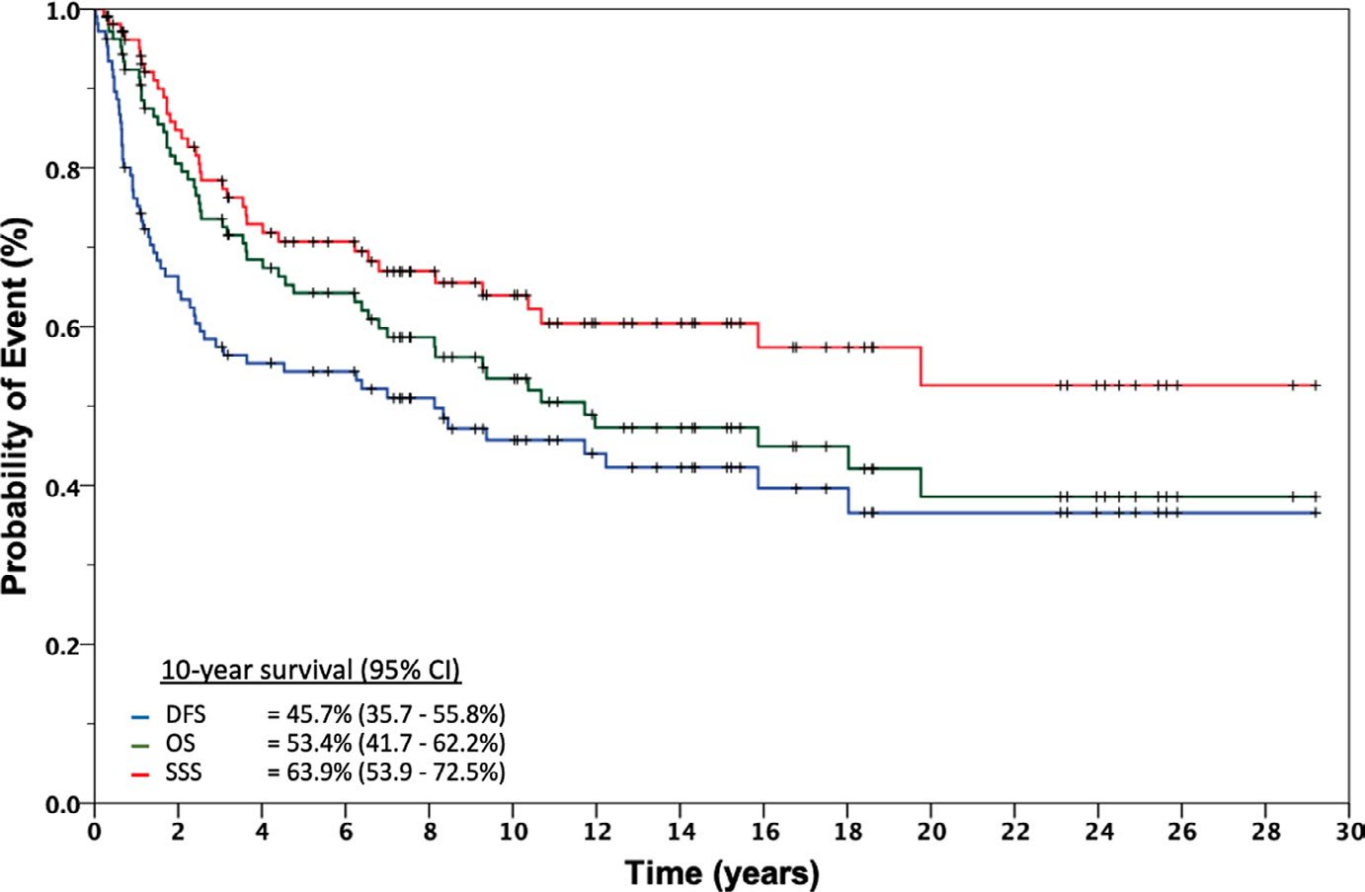
Disease‐free, overall, and sarcoma‐specific survival for NONE‐STS of bone treated from 1987 to 2017. Abbreviations: DFS, disease‐free survival; OS, overall survival; SSS, Sarcoma‐specific survival; NONE, non‐osteosarcoma, non‐Ewing

Median follow‐up for the full cohort of 106 patients was 17.45 years (range 1.73 to 33.27) (Figure 2). In the full cohort, the 10‐year DFS was 45.7% (95%CI: 35.7‐55.8%), OS was 53.4% (95%CI: 41.7‐62.2%), and SSS was 63.9% (95%CI: 53.9‐72.5%). The median DFS was 8.1 years (95% CI: 2.5‐18.0), OS was 11.7 years (95%CI: 4.5‐18.9), and was not reached for SSS (Figure 3).

**FIGURE 4:**
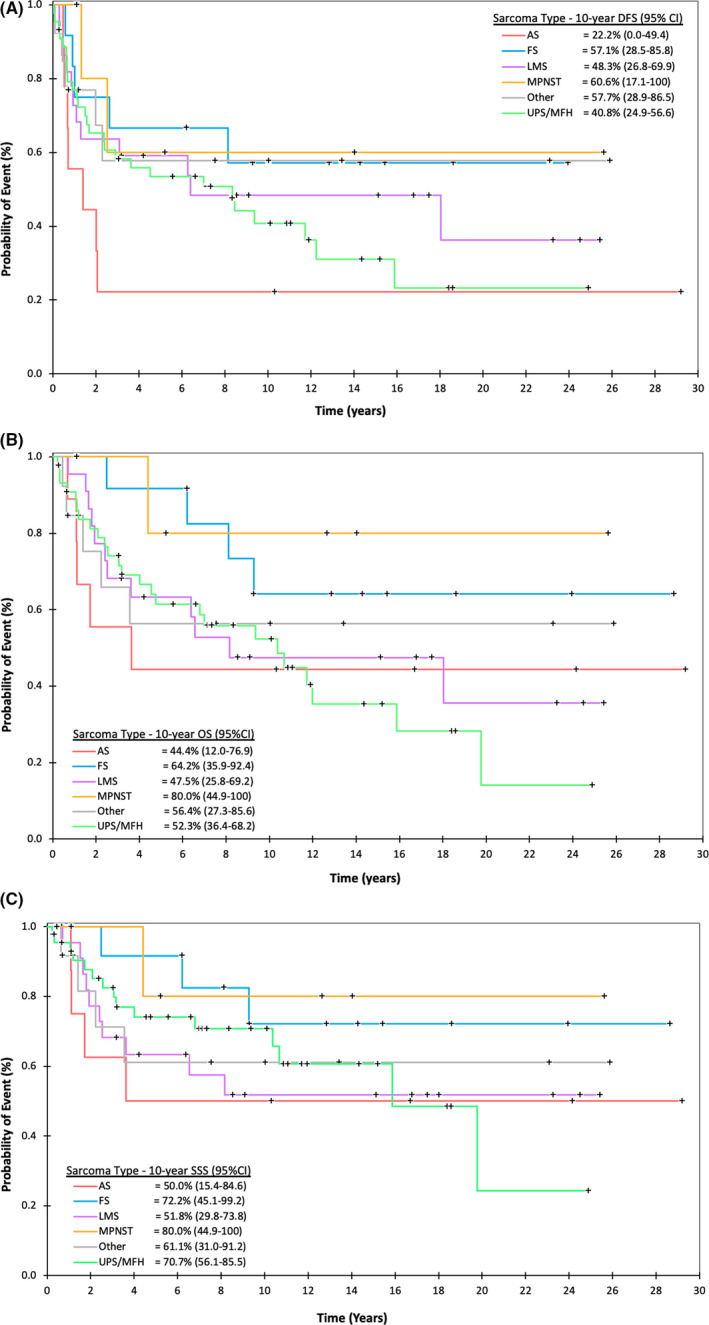
Disease‐free (A), overall (B), and sarcoma‐specific survival (C) for NONE‐STS of bone by histology. Abbreviations: AS, angiosarcoma; FS, fibrosarcoma; LMS, leiomyosarcoma; MPNST, malignant peripheral nerve sheath tumor; UPS, undifferentiated pleomorphic sarcoma; MFH, malignant fibrous histiocytoma; NONE, non‐osteosarcoma, non‐Ewing

Evaluation of 10‐year DFS, OS, and SSS, respectively, by sarcoma histology (Figure 4A/B/C) identified MPNST as having the most favorable prognosis (DFS = 60.6%, [95%CI: 17.1‐100%]; OS = 80.0%, [95%CI: 44.9‐100%]; SSS = 80.0%, [95%CI: 44.9‐100%]); albeit in a small group of only six patients. Conversely, AS was associated with one of the least favorable long‐term prognoses (DFS = 22.2%, [95%CI: 0.0‐49.4%]; OS = 44.4%, [95%CI: 12.0‐76.9]; SSS = 50%, [95%CI: 15.4‐84.6]). Survival for most sarcoma histologies plateaued after 10 years of follow‐up, with the exception of UPS/MFH which demonstrated further sarcoma‐related death beyond 10 years (SSS at 5 year [74.1%], 10 year [70.7%], 15 year [60.6%], and 20 year [24.2%]) (Figure 4C and Appendix 1).

### 3.3 Univariate and multivariate analysis

Evaluation of patient and treatment characteristics using SSS in univariate analysis (Table 2A) identified high grade (HR = 8.03, [95%CI: 1.09‐59.36]; *p* = 0.041) and disease recurrence (HR = 90.43, [95%CI: 12.34‐662.88]; *p* < 0.001) with higher risk of sarcoma‐related death. Patients treated with neoadjuvant chemotherapy who achieved 50‐85% treatment‐induced tumor necrosis (HR = 0.22, [95%CI: 0.05‐0.96]; *p* = 0.044), and those with postoperative complications (HR = 0.37, [95%CI: 0.15‐0.84]; *p* = 0.017) were associated with lower risk of sarcoma‐related death. Adjustment for confounders in multivariate analysis (Table 2B) identified axial location (HR = 35.50, [95%CI: 3.41‐369.60]; *p* = 0.003), intermediate (HR = 16.93, [95%CI: 1.10‐261.24]; *p* = 0.043) or high (HR = 16.94, [95%CI: 1.55‐185.07]; *p* = 0.020) grade, and disease relapse (HR = 485.09, [95%CI: 36.30‐6482.60]; *p* < 0.001) as significant risk factors for sarcoma‐related death. Characteristics associated with lower risk of sarcoma‐related death in multivariate analysis were tumor size >10 cm (HR=0.19, [95%CI: 0.32‐2.85]; *p* = 0.050), receipt of chemotherapy (HR = 0.07, [95%CI: 0.01‐0.86]; *p* = 0.037), and tumor necrosis ≥85% (HR = 0.20, [95%CI: 0.04‐0.99]; *p* = 0.048).

**TABLE 2:** Univariate (A) and multivariate (B) Cox proportional hazards analyses of clinical characteristics and treatment‐related factors for sarcoma‐specific survival in NONE‐STS of bone

Characteristic	Univariate (A)			Multivariate (B)		
	HR	95% CI	*p*‐value	HR	95% CI	*p*‐value
**Age**						
<50	**REF**		**REF**			
≥50	1.66	0.87‐3.17	0.127	2.98	0.80‐11.14	0.106
**Sex**						
Male	**REF**		**REF**			
Female	0.89	0.46‐1.73	0.722	0.41	0.14‐1.24	0.114
**Histology**						
UPS/MFH	**REF**	0.740	**REF**	0.050		
Leiomyosarcoma	1.21	0.54‐2.71	0.628	0.47	0.11‐2.53	0.430
Fibrosarcoma	0.53	0.15‐1.82	0.321	0.36	0.05‐2.42	0.292
Angiosarcoma	1.36	0.45‐4.13	0.587	0.28	0.04‐2.13	0.221
MPNST	0.41	0.05‐3.08	0.383	0.17	0.01‐4.32	0.281
Other	1.04	0.34‐3.13	0.949	5.74	0.79‐42.03	0.09
**Location**						
Lower	**REF**	0.358	**REF**	<0.001		
Upper	0.23	0.03‐1.71	0.152	0.15	0.01‐1.71	0.126
Axial	0.92	0.28‐3.01	0.894	35.50	3.41‐369.60	**0.003**
**Tumor Size**						
≤5 cm	**REF**	0.844	**REF**	0.201		
>5‐10 cm	0.90	0.33‐2.45	0.832	0.42	0.11‐1.65	0.213
>10 cm	0.96	0.32‐2.85	0.935	0.19	0.04‐1.00	**0.049**
Unknown	1.35	0.43‐4.24	0.613	0.19	0.03‐1.47	0.112
**Grade**						
1	**REF**	0.145	**REF**	0.145		
2	5.17	0.62‐43.00	0.128	16.93	1.10‐261.24	**0.043**
3	8.03	1.09‐59.36	**0.041**	16.94	1.55‐185.07	**0.020**
Unknown	10.60	1.17‐95.91	**0.036**	9.13	0.65‐128.12	0.100
**Chemotherapy**						
No	**REF**		**REF**			
Yes	0.90	0.45‐1.80	0.770	0.07	0.01‐0.86	**0.037**
**Postoperative Complications**						
No	**REF**		**REF**			
Yes	0.37	0.16‐0.84	**0.017**	0.53	0.15‐1.83	0.313
**Disease Recurrence**						
No	**REF**		**REF**			
Yes	90.43	12.34‐662.88	**<0.001**	485.09	36.30‐6482.60	**<0.001**
**Necrosis**						
0‐49%	**REF**	0.103	**REF**	0.198		
50‐84%	0.22	0.05‐0.96	**0.044**	2.01	0.30‐13.67	0.475
85‐100%	0.45	0.16‐1.26	0.130	0.20	0.04‐0.99	**0.048**
Unknown	1.28	0.42‐3.91	0.690	0.29	0.03‐2.54	0.260
No Chemotherapy	0.54	0.25‐1.17	0.119	0.32	0.02‐4.05	0.375
**Radiation**						
No	**REF**		**REF**			
Yes	0.86	0.21‐3.57	0.830	0.28	0.04‐2.12	0.219

Abbreviations: MFH, malignant fibrous histiocytoma; MPNST, malignant peripheral nerve sheath tumor; NONE, non‐osteosarcoma, non‐Ewing; UPS, undifferentiated pleomorphic sarcoma.

## 4 DISCUSSION

In this study of NONE‐STS of bone, we demonstrate key prognostic findings of soft‐tissue sarcoma histologies with rare presentations as bone tumors. Intrinsic to this dataset is consistency in treatment approach with multidisciplinary tumor board discussion, expert pathologic, and radiographic review at a quaternary referral center.

At present, this study represents the largest group of patients with localized NONE‐STS of bone treated with curative intent. Consistent with other studies of soft‐tissue and osteosarcoma, high degree of necrosis on final pathology^18^, ^19^, ^20^ and receipt of chemotherapy^22^, ^23^ were associated with improved survival; whereas axial tumor location^24^ and high pathologic grade^1^ were associated with worse outcomes on multivariate analyses. Our data further demonstrates favorable 5‐year (70.7%) and 10‐year (63.9%) SSS for the full cohort of 106 patients, with the notable finding of sarcoma‐related death for UPS/MFH beyond 10 years (Appendix 1). Previous studies by Berner et. al using Norwegian Cancer Registry data demonstrated moderate 5‐year SSS (45%) for spindle cell sarcomas (LMS, UPS, and FS) of bone, with subgroup analysis of UPS showing a 5‐year SSS of 37%, with recurrence out to 10 years.^1^ Additional studies by Nishida et al, have also shown poor survival trends for UPS of bone at 2‐ (61%), 5‐ (53%), and 10‐years (41%) post‐surgery.^8^ Differences in study inclusion criteria (e.g.: metastatic^1^ vs non‐metastatic^8^), and pathologic evaluation following the reclassification of MFH to UPS^25^ may account for survival variance between studies. Despite this, all three publications demonstrate relapse for the UPS subtype at 10 years and beyond. This finding could be used to guide clinical discussions and may warrant longer surveillance for patients diagnosed with UPS of bone.

Survival outcomes for nonmetastatic FS of bone with modern treatment techniques is generally unknown. In this study, we identified high 5‐ (92%), and 10‐year (72%) SSS in this cohort of 12 patients (Appendix 1). In the Norwegian Cancer Registry study 5‐year SSS was 15% for fibrosarcoma in subgroup analysis^1^; however, this analysis included patients with metastatic disease, which likely contributed to poor outcomes. Survival of patients with LMS and AS of bone in our cohort was similar to results documented in other studies^10^, ^13^. MPNST is a rare type of soft tissue sarcoma that can occur sporadically or in association with Neurofibromatosis type 1 (NF‐1), an autosomal dominant condition characterized by development of peripheral nerve sheath tumors called neurofibromas.^26^ As MPNST of bone has only been described in case reports, here we report high 10‐year OS (80%) and SSS (80%), although in a small group of only six patients.

This study has a number of limitations intrinsic to its retrospective nature and the time period over which it was collected (30 years). With respect to unknown or missing data, this was as high as 29.2% in some categories (e.g.: number of chemotherapy cycles). For characteristics included in univariate and multivariate analyses, misclassification bias may have affected some categories such as unknown grade (N = 10; 9.4%), which may have led to a chance finding on univariate analysis. This may have also been a factor in the finding of improved survival associated with tumors >10 cm in size by multivariate analysis, as (reason would argue) these patients should have worse outcomes. Yet, adding to the robustness of this analysis, many other factors associated with improved survival (e.g.: administration of chemotherapy, >85% tumor necrosis) or worse outcomes (e.g.: higher grade, pelvic tumor location, and disease relapse) remained biologically plausible, and significant in analyses.

A further limitation of this study is related to the increased stringency in the classification of UPS over the last 3 decades.^25^ As no retrospective pathologic review was carried out for patients with UPS/MFH in this study, this may have led to misclassification bias of other sarcoma subtypes in the UPS/MFH category. Additionally, given the rarity of NONE‐STS of bone, specific subgroups have small patient numbers, possibly leading to imprecise survival estimates.

In conclusion, NONE‐STS of the bone remains a rare diagnosis. This study identifies grouped‐ and tumor‐specific survival and risk factors that inform clinical discussions and decisions in the modern treatment era.

## CONFLICT OF INTEREST

**ZWV** has received honorarium from Pfizer and Genomic Health. He has also served on an advisory board for Genomic Health. **ARAR** has received research funding from Roche, Genentech, Eli Lilly, Merck, Boehringer Ingelheim, Novartis, AbbVie, Deciphera, Karyopharm, Astra Zeneca, Medimmune, Blueprint, Bristol Myers Squibb, GSK, Entremed/Casi Pharmaceuticals, Adaptimmune, and BetaCat. He has also served the advisory board for Eli Lilly, Merck, Adaptimmune, and Boehringer Ingelheim. The following authors have declared no conflicts of interest: AG, SF, EMA, AAG, PF, and JSW.

## AUTHOR CONTRIBUTIONS

Conceptualization—ZWV, JSW, and ARAR. Data curation—ZWV and AG. Formal Analyses—ZWV. Funding acquisition—None. Investigation—ZWV, ARAR, and JSW. Methodology—ZWV, AAG, and ARAR. Project Administration—ARAR. Resources‐ AAG and JSW. Software—ZWV. Supervision—ARAR, JSW, and AG. Validation—Not applicable. Visualization—ZWV. Writing—Original Draft—ZWV. Writing—Review and Editing—ZWV, AG, SF, EMA, AAG, PF, JSW, and ARAR.

## Data Availability

The data that support the findings of this study are available on request from the corresponding author. The data are not publicly available due to privacy or ethical restrictions.

## Appendix 1 Survival by year for NONE bone sarcoma subtypes

**Table:**

Disease‐free	5 year (%)	10 year (%)	15 year (%)
Angiosarcoma	22.2	22.2	22.2
Fibrosarcoma	66.7	57.1	57.1
LMS	59.1	48.3	48.3
MPNST	60.0	60.0	60.0
Other	57.7	57.7	57.7
UPS/MFH	53.4	40.8	31.1
**Overall**			
Angiosarcoma	44.4	44.4	44.4
Fibrosarcoma	91.7	64.2	64.2
LMS	63.3	47.5	47.5
MPNST	80.0	80.0	80.0
Other	56.4	56.4	56.4
UPS/MFH	61.4	52.3	35.3
**Sarcoma specific**			
Angiosarcoma	50.0	50.0	50.0
Fibrosarcoma	91.7	72.2	72.2
LMS	63.3	51.8	51.8
MPNST	80.0	80.0	80.0
Other	61.1	61.1	61.1
UPS/MFH	74.1	70.7	60.6

Abbreaviations: MFH, malignant fibrous histiocytoma; MPNST, malignant peripheral nerve sheath tumor; NONE, non‐osteosarcoma, non‐Ewing; UPS, undifferentiated pleomorphic sarcoma.
